# Supplementary material for: Diagnostic SARS-CoV-2 Cycle Threshold Value Predicts Disease Severity, Survival, and Six-Month Sequelae in COVID-19 Symptomatic Patients
Source: Viruses. 2021 Feb 11;13(2):281. doi: 10.3390/v13020281 (PMC7917896; doi:10.3390/v13020281)
Supplement: Supplementary file 1 [file viruses-13-00281-s001.pdf]

viruses

MDPI

Supplementary Material

<table>
<tr><td>Patient Identificative Code</td><td colspan="6"></td><td colspan="2">PCR Ct</td><td colspan="2"></td></tr>
<tr><td>First positive swab (date)</td><td></td><td>Age</td><td colspan="4" rowspan="2"></td><td colspan="2">Hospital Admission</td><td>Yes</td><td>No</td></tr>
<tr><td>Sex</td><td>M</td><td>F</td><td>Outcome</td><td>Complete recovery</td><td>Death</td><td>Sequelae (specify)</td></tr>
<tr><td>Confirmative negative swab (date)</td><td></td><td>Oxygen support</td><td>No</td><td>Yes</td><td>CPAP</td><td>Intub</td><td>Comorbid.</td><td>Diabetes</td><td>Hypertension</td><td>Overweight/obesity</td></tr>
<tr><td colspan="7"></td><td>Asthma</td><td>COPD</td><td>Cancer</td><td>Act. Smoking</td></tr>
<tr><td>Time from COVID-19 onset and first swab (days)</td><td colspan="6"></td><td colspan="4">Other (specify)</td></tr>
<tr><td>Signs and Symptoms at the diagnosis</td><td colspan="10"></td></tr>
<tr><td>Notes</td><td colspan="10"></td></tr>
</table>

Figure S1. Phone survey questionnaire.

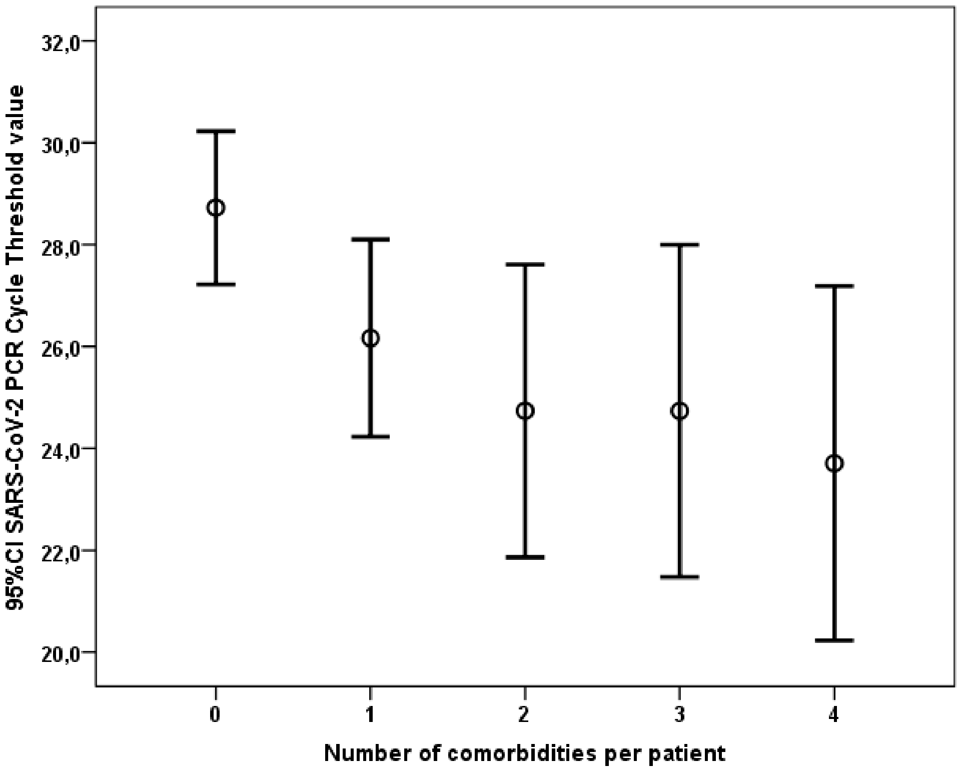

Figure S2. Distribution of SARS-CoV-2 PCR Cycle threshold values according to the number of comorbidities per patient at COVID-19 diagnosis.

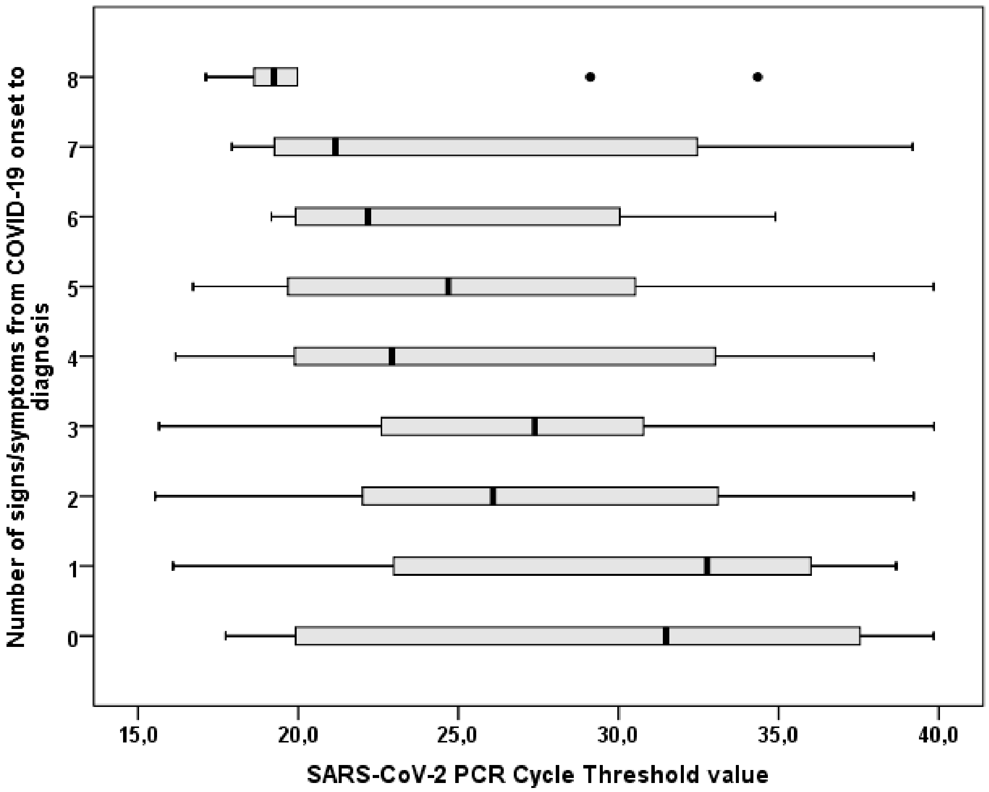

Figure S3. Distribution of SARS-CoV-2 PCR cycle threshold values according to the number of signs and symptoms of COVID-19 at diagnosis.
